# Basic life support knowledge of secondary school students in cardiopulmonary resuscitation training using a song

**DOI:** 10.5116/ijme.5780.a207

**Published:** 2016-07-20

**Authors:** Francisco Javier Fonseca del Pozo, Joaquin Valle Alonso, Nancy Beatriz Canales Velis, Mario Miguel Andrade Barahona, Aidan Siggers, Elisa Lopera

**Affiliations:** 1 Emergency and Critical Care Unit of Montoro (Córdoba), Instituto Maimónides de Investigación Biomédica de Córdoba, Spain; 2 Department of Emergency Medicine, Royal Bournemouth and Christchurch NHS Foundation Trust, UK; 3 Hospital Comarcal de la Selva, Blanes, Girona, Spain; 4 Department of Emergency Medicine, Hospital Comarcal Valle de Los Pedroches. Pozoblanco (Córdoba), Spain

**Keywords:** CPR training, school adolescents, compressions, song

## Abstract

**Objectives:**

To examine the effectiveness of a “cardiopulmonary resuscitation song” in improving the basic life support skills of secondary school students.

**Methods:**

This pre-test/post-test control design study enrolled secondary school students from two middle schools randomly chosen in Córdoba, Andalucia, Spain. The study included 608 teenagers. A random sample of 87 students in the intervention group and 35 in the control group, aged 12-14 years were selected. The intervention included a cardiopulmonary resuscitation song and video. A questionnaire was conducted at three-time points: pre-intervention, one month and eight months post-intervention.

**Results:**

On global knowledge of cardiopulmonary resuscitation, there were no significant differences between the intervention group and the control group in the trial pre-intervention and at the month post-intervention. However, at 8 months there were significant differences with a p-value = 0.000 (intervention group, 95% CI: 6.39 to 7.13 vs. control group, 95% CI: 4.75 to 5.92), (F _(1,120)_=16.644, p= 0.000). In addition, significant differences about students’ basic life support knowledge about chest compressions at eight months post-intervention (F_(1,120)_=15.561, p=0.000) were found.

**Conclusions:**

Our study showed that incorporating the song component in the cardiopulmonary resuscitation teaching increased its effectiveness and the ability to remember the cardiopulmonary resuscitation algorithm. Our study highlights the need for different methods in the cardiopulmonary resuscitation teaching to facilitate knowledge retention and increase the number of positive outcomes after sudden cardiac arrest.

## Introduction

Cardiac arrest is an immediate medical emergency, which can be effectively managed if effective cardiopulmonary resuscitation is commenced promptly. Recent studies show that bystander cardiopulmonary resuscitation (CPR) is the key factor for determining out of hospital cardiac arrest survival.^1^^,^^2^ It is, therefore, critical to educate young people in CPR as a long-term strategy for the community. Training school children to perform cardiopulmonary resuscitation is one possible method of increasing bystander CPR rates. Evidence shows that cardiopulmonary training, delivered in various ways, is successful in a wide age range of children.^3^^-^^16^ Studies performed over a wide period and looking at a variety of approaches to training schoolchildren in CPR and associated skills show that all training interventions are successful within a short time scale in increasing knowledge and skills of children when tested.^17^ While older children perform more successfully on testing, younger children can perform basic tasks well, including the use of automated external defibrillator. The most efficient method is however not well established.^17^

A fundamental problem in learning and performing CPR manoeuvres is the loss of acquired knowledge over time. In one study looking at the retention of CPR skills of successfully trained adults, after 12 months only 47% demonstrated correct hand placement on the manikin; 44% adequately performed the depth of compressions and compression rate was adequate in just 59%.^18^

One of the methods used in the training of bystanders in CPR is student participation in training courses. A Norwegian study demonstrated that CPR training can be disseminated in a population by distributing personal resuscitation manikins among children in primary schools.^19^ The teachers felt able to easily facilitate CPR training although the incidence of bystander CPR did not increase significantly in the months following the project.  In a study by Jones et al,^14^only children from 13 to 14 and older performed chest compressions as well as adults. In order to achieve the depth of chest compressions for adult in cardiac arrest, it requires the application of about 50_kg_. Therefore, it seems reasonable to limit the practice of skills for compressing the chest to children over 13 years.

Chest compression depth correlates with physical factors such as increasing weight, body mass index and height. Instruction must include hands-on practice to enable children to perform physical tasks. Repeated training improves performance and retention, but the optimal form and frequency of repeated training are yet to be fully determined. Training interventions should be age-appropriate and practical and should both reinforce core ideas and sequentially introduce skills of greater complexity. Brevity, diversity of format and attention to cost and efficiency will promote interest from children and schools. Types of training that may reduce the main obstacles to the implementation of such training in schools include the use of self-instruction kits, computer-based learning and use of teacher and peer tutor trainers, but again, further exploration is required.^19^

This study assessed the basic life support knowledge and performance of secondary school students before and after CPR training with a song and video that was specifically created for this purpose by local authors, with special focus on compressions and easy to remember.

## Methods

### Study design

Between the periods of January 2013 to January 2014, we conducted a pre-test/post-test control design study with geographical convenience sampling from two populations of secondary school students with similar characteristics in the province of Cordoba (Spain).

### Sample size and sampling procedures

A total sample size 608 students (306 females), aged between 12 to 14 years of the first and second year of secondary education was arbitrarily and conveniently defined (438 students from the intervention place, and 170 from control place). A weighted stratified sampling procedure was applied according to each of the classes. The consent of all parents was obtained after an explanation of nature and the purpose of the study. None of the students knew the song before the study. Students’ participation was on a voluntary basis; students who did not attend the workshops suffered any disability that does not allow participating in the workshops and students that did not have the signature of the informed consent of their parents or guardians were excluded. The study protocol and data collection instrument were reviewed and approved by the Ethics Committee of the University Hospital Reina Sofía (Córdoba).

**Table 1:** Comparative table of the overall average score of both groups in pre-intervention and post-intervention test after one month and eight month intervals

Score	Type of intervention	Average	Standard deviation	CI 95%	
				lower	upper
Pre- intervention	Song	4.48	1.97	4.08	4.88
	No song	4.19	1.64	3.56	4.82
Month post- intervention	Song	6.44	1.49	6.10	6.77
	Traditional	5.90	1.78	5.38	6.43
8 months post- intervention	Song	6.76	1.69	6.39	7.13
	Traditional	5.33	1.89	4.75	5.92

### Data collection method and procedure

For this study, a song was created following the recommendations of the International Liasion Committee on Resuscitation ILCOR 2010.^20^   The song was titled "You can" and has been recorded in the General Society of Authors and Editors. The content focuses on knowing what the sequence of CPR is, such as the recognition of unconsciousness, calling the emergency number 112, starting chest compressions and ventilations and a chorus where the idea of managing cycles of 30 compressions and two ventilations is repeated.

**Table 2:** Comparative table showing the average quality of compressions in both groups pre- and post-intervention

Score	Type of intervention	Average	Standard deviation	CI 95%	
				lower	upper
Pre- intervention	Song	3.03	2.67	2.47	3.58
	Traditional	3.24	2.49	2.36	4.12
Month post- intervention	Song	7.13	1.98	6.68	7.58
	Traditional	6.19	2.44	5.48	6.90
8 months post- intervention	Song	7.01	2.44	6.48	7.54
	Traditional	5.05	2.60	4.22	5.88

To assess the acquisition of theoretical knowledge we used a previously validated multiple choice CPR questionnaire based on PROCES study.^21^ The structured questionnaire contained eight questions based on practical CPR, evaluating the main features of the technique. Each question had four possible answers, of which only one was correct. Prior validation was performed in a total of 50 students, introducing the changes suggested by them, mainly regarding understanding; once changes were done, the final questionnaire was developed. The questions evaluated included; Recognition of unconsciousness, initial evaluation, ratio of compressions/ventilations, characteristics of effective compressions, recognizing the phone number of emergency medical services (EMS), after calling EMS to report a cardiac arrest, what should you do, correct position for the administration of compressions and rescuers switch roles when performing 2-rescuer CPR.

**Figure 1:**
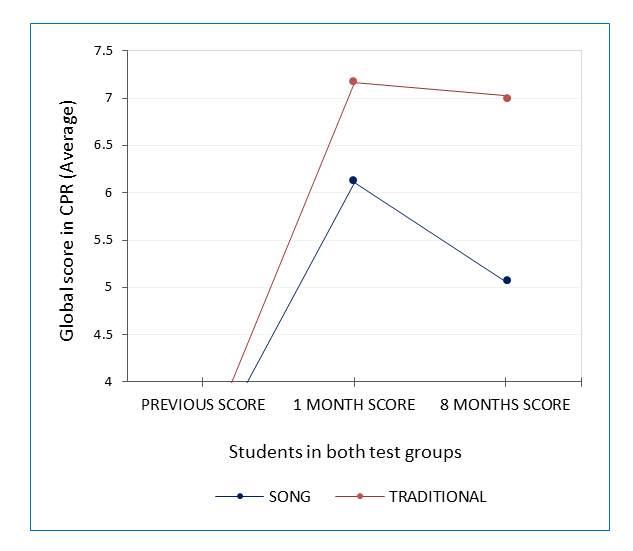
Comparative analysis of the average scores obtained by students in both test groups in pre-intervention and post-intervention at one month and eight months

The scoring system was 1 point per correct question. The total available mark was 8 points. The test was considered passed it the student was able to give 6 correct answers. After completing the initial questionnaire, students in the intervention group participated in a workshop of basic CPR for the general population. It explained the importance of scene safety, the proper position of the victim to perform CPR, recognition of unconsciousness, opening the airway, calling emergency service via the number 112, start of chest compressions, placement of the arms and hands, cardiac massage point location and depth and frequency. It highlighted the importance of giving continued chest compressions without interruptions, mouth to mouth ventilation, 30:2 thoracic compressions and ventilation relation and when to suspend cardiac massage.

Upon completion, students sang the same song and learned a specific choreography-oriented to CPR techniques. Students in the control group were taught basic CPR course for the general population; the main difference was that the song was not applied to reinforce learned skills. To assess retention of knowledge students completed the same questionnaire at one month and eight months after the intervention.

### Statistical analysis

Questionnaire data were introduced into a database program SPSS 15.0. A first analysis using data from the completed questionnaire and a second analysis with the answers regarding chest compressions were performed, about the ILCOR recommendations in promoting CPR for the training of uninitiated rescuers. The results are shown in tables and graphics. To compare the results between the two groups, analysis of variance for repeated measures has been applied. Statistical significance was set at p < 0.05 and confidence intervals of 95% (95% CI) of the mean differences.

## Results

The study included a total of 370 students in the intervention group and 128 in the control group. A single representative random sample of 87 from the intervention group and 35 students from the control group were obtained, students who had not done any of the three questionnaires were not included.

The score in pre-test workshops had a homogeneous score since the intervention group had an average score of 4.48 (95% CI: 4.08 to 4.88), similar to the control group average score 4.19 (95% CI: 3.56 to 4.82). With regard to the assessment of knowledge at one and eight months, students in the control group went to an average score of 5.9 (95% CI: 5.38 to 6.43) to 5.33 (95% CI: 4.75 to 5.92) compared to students in the intervention group that increased from 6.44 (95% CI: 6.10 to 6.77) to 6.76 (95% CI: 6.39-7.13) (Table 1).

Both the pre-intervention questionnaire and the questionnaire performed one month after the intervention showed no significant differences between the two groups; however, at 8 months significant difference was shown with a value of p = 0.000 (Intervention 95% CI: 6.39 to 7.13 vs control 95% CI: 4.75 to 5.92), (F _(1,120)_=16.644, p= 0.000), Figure 1.

**Figure 2:**
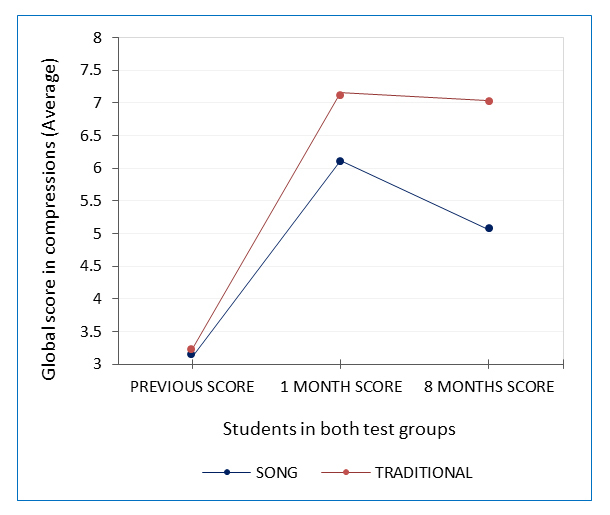
Comparative analysis showing the average quality of compressions related to both groups pre- and post-intervention

Taking into account the recommendations of prioritizing compressions in bystander-only CPR, answers were rated regarding the quality of chest compressions, relationship to ventilations and the hand position. Table 2 shows the results. We can see that although average scores are very similar pre-intervention, there are marked differences between groups at one month and eight months. In Figure 2, the differences are significant (F _(1, 120 _= 15.561, p= 0.000), Figure 2.

## Discussion

The aim of the study was to examine the effectiveness of a CPR song in improving the secondary grade students skills in BLS. After eight months, a significant difference was obtained in the group of secondary grade students that were trained with the CPR song and video.

Studies assessing the success of training courses demonstrate significant improvements in performance after training, compared to baseline, in children of all ages, from 4 to 20 years.^17^ Increasing the percentage of the population trained in CPR is an integral part of an overall strategy to improve community response to the Out-of-hospital cardiac arrest. Schools provide excellent access to a large part of the community. Over time, a significant percentage of the overall community will receive training. Programs in which students can share materials used in school-based programs at home with family members can further increase the program’s yield regarding the total number of members of the community trained per unit of class time expended.^19^^,^^22^ In this work we demonstrate that use of a song related to basic CPR is capable of reducing the loss of acquired theoretical knowledge compared to methods using only the traditional basic CPR training. Taking into account the ILCOR recommendation to prioritize thoracic compressions,^20^ we can see the effectiveness of this new tool to embed knowledge, as the lyrics and choreography used encouraged learning in a fun manner, fulfilling the premise that it is easier to remember the lyrics of a song that the theory learned in a workshop.^23^  Previous studies have used popular songs like “Macarena” by Los del Río, Staying Alive and Nellie the elephant to improve the quality of compressions, especially the frequency of compressions. Unlike them, the song “You can” has a content based not only on chest compressions but on the entire sequence of basic CPR. Further work is needed to check if the song is effective in preserving acquired skills in the performance of CPR, in addition to improving the quality of compressions.

The results demonstrate the effectiveness of a new tool to reinforce the theoretical knowledge acquired on CPR, using a song whose lyrics facilitates learning by school adolescents. An advantage of this study is the limited geographical mobility of the group, a difficult obstacle to overcome in large cities that has affected monitoring in other studies.

CPR instruction including the use of automatic external defibrillators has shown to be feasible even for young schoolchildren; earlier training may lead to better results.

The programme 'Kids Save Lives' recently endorsed by the WHO aims to help promote schools-based BLS training worldwide, requiring education on CPR for all pupils starting at least at age 12. We believe that the use of different teaching methods similar to the exposed in our work may encourage students to retain knowledge in CPR.

### Limitations

The present study has a limitation in that it was performed on a small sample collected from a single location. Therefore further research on this topic is warranted using a larger sample.  Our study investigated secondary students over an eight moth period only. It is unknown whether the students can retain the acquired knowledge over a longer period. It would, therefore, be appropriate to repeat the knowledge test a year or longer post-intervention. It is important to compare the results with residents in other areas, such as large cities, as they have different sociodemographic characteristics.

## Conclusions

We may conclude that this method of basic life support training in secondary schools students seems highly effective.  We observed significant improvement and a good retention rate eight months after training in the intervention group. Our study showed that incorporating the song component in the CPR education increases its effectiveness. The use of a song and a video may show promise in the teaching of CPR in secondary school students and should be considered in the future training of this group.

### Conflict of Interest

The authors declare that they have no conflict of interest.
